# APOE ε4 genotype-dependent cerebrospinal fluid proteomic signatures in Alzheimer’s disease

**DOI:** 10.1186/s13195-020-00628-z

**Published:** 2020-05-27

**Authors:** Elles Konijnenberg, Betty M. Tijms, Johan Gobom, Valerija Dobricic, Isabelle Bos, Stephanie Vos, Magda Tsolaki, Frans Verhey, Julius Popp, Pablo Martinez-Lage, Rik Vandenberghe, Alberto Lleó, Lutz Frölich, Simon Lovestone, Johannes Streffer, Lars Bertram, Kaj Blennow, Charlotte E. Teunissen, Robert Veerhuis, August B. Smit, Philip Scheltens, Henrik Zetterberg, Pieter Jelle Visser

**Affiliations:** 1 Alzheimer Center Amsterdam, Department of Neurology, Amsterdam Neuroscience, Amsterdam UMC, Vrije Universiteit Amsterdam, PO Box 7057, 1007 MB Amsterdam, The Netherlands; 2 Clinical Neurochemistry Laboratory, Sahlgrenska University Hospital, Mölndal, Sweden; 3 Department of Psychiatry and Neurochemistry, Institute of Neuroscience and Physiology, Sahlgrenska Academy at the University of Gothenburg, Mölndal, Sweden; 4 Lübeck Interdisciplinary Platform for Genome Analytics, University of Lübeck, Lübeck, Germany; 5 Department of Psychiatry and Neuropsychology, School for Mental Health and Neuroscience, Alzheimer Centre Limburg, Maastricht University, Maastricht, The Netherlands; 6 1st Department of Neurology, AHEPA University Hospital, Thessaloniki, Macedonia Greece; 7 Department of Psychiatry, University Hospital of Lausanne, Lausanne, Switzerland; 8 Department of Neurology, Center for Research and Advanced Therapies, CITA-Alzheimer Foundation, San Sebastian, Spain; 9 University Hospital Leuven, Leuven, Belgium; 10 Hospital de la Santa Creu i Sant Pau, Barcelona, Spain; 11 Department of Geriatric Psychiatry, Zentralinstitut für Seelische Gesundheit, University of Heidelberg, Mannheim, Germany; 12 Department of Psychiatry, University of Oxford, Oxford, UK; 13 Janssen R&D, Beerse, Belgium; 14 Early Clinical Neurology, UCB Biopharma SPRL, Braine-l’Alleud, Belgium; 15 Present Address: Janssen R&D, LLC, Beerse, Belgium; 16 School of Public Health, Imperial College London, London, UK; 17 Department of Psychology, University of Oslo, Oslo, Norway; 18 Clinical Neurochemistry Lab, Institute of Neuroscience and Physiology, Sahlgrenska University Hospital, Mölndal, Sweden; 19 Department of Psychiatry and Neurochemistry, Institute of Neuroscience and Physiology, University of Gothenburg, Mölndal, Sweden; 20 Neurochemistry Laboratory and Biobank, Department of Clinical Chemistry, Amsterdam Neuroscience, Amsterdam UMC, Vrije Universiteit Amsterdam, Amsterdam, The Netherlands; 21 Department of Psychiatry, Amsterdam Neuroscience, Amsterdam UMC, Vrije Universiteit Amsterdam, Amsterdam, The Netherlands; 22 Department of Molecular and Cellular Neurobiology, Center for Neurogenomics and Cognitive Research, Amsterdam Neuroscience, VU University Amsterdam, Amsterdam, The Netherlands; 23 Department of Molecular Neuroscience, UCL Institute of Neurology, London, UK; 24 UK Dementia Research Institute, London, UK; 25 Department of Neurobiology, Care Sciences and Society, Division of Neurogeriatrics, Karolinska Instutet, Stockholm, Sweden

**Keywords:** Amyloid aggregation, APOE genotype, CSF proteomics

## Abstract

**Background:**

Aggregation of amyloid β into plaques in the brain is one of the earliest pathological events in Alzheimer’s disease (AD). The exact pathophysiology leading to dementia is still uncertain, but the apolipoprotein E (APOE) ε4 genotype plays a major role. We aimed to identify the molecular pathways associated with amyloid β aggregation using cerebrospinal fluid (CSF) proteomics and to study the potential modifying effects of APOE ε4 genotype.

**Methods:**

We tested 243 proteins and protein fragments in CSF comparing 193 subjects with AD across the cognitive spectrum (65% APOE ε4 carriers, average age 75 ± 7 years) against 60 controls with normal CSF amyloid β, normal cognition, and no APOE ε4 allele (average age 75 ± 6 years).

**Results:**

One hundred twenty-nine proteins (53%) were associated with aggregated amyloid β. APOE ε4 carriers with AD showed altered concentrations of proteins involved in the complement pathway and glycolysis when cognition was normal and lower concentrations of proteins involved in synapse structure and function when cognitive impairment was moderately severe. APOE ε4 non-carriers with AD showed lower expression of proteins involved in synapse structure and function when cognition was normal and lower concentrations of proteins that were associated with complement and other inflammatory processes when cognitive impairment was mild. Repeating analyses for 114 proteins that were available in an independent EMIF-AD MBD dataset (*n* = 275) showed that 80% of the proteins showed group differences in a similar direction, but overall, 28% effects reached statistical significance (ranging between 6 and 87% depending on the disease stage and genotype), suggesting variable reproducibility.

**Conclusions:**

These results imply that AD pathophysiology depends on APOE genotype and that treatment for AD may need to be tailored according to APOE genotype and severity of the cognitive impairment.

## Background

Amyloid β aggregation in the brain is one of the earliest pathological events in Alzheimer’s disease (AD) and is thought to start decades before the manifestation of dementia [1–3]. The presence of an apolipoprotein E (APOE) ε4 allele, the major genetic risk factor for AD [4], lowers the age of onset through an as of yet unknown mechanism. In general, it is largely unclear which biological processes eventually lead to cognitive decline once amyloid β has aggregated, as well as whether such processes are influenced by the presence of the APOE ε4 allele. A better understanding of the biological processes disrupted in AD subjects is crucial for the development of precision medicine. The apoE4 protein isoform has been associated with impaired amyloid clearance and transport, synaptogenesis, and glucose and cholesterol metabolism in the brain [5, 6]. However, about 25–40% of patients with AD dementia lack the APOE ε4 allele [7], and for these individuals, the pathophysiological mechanisms involved in AD are less clear [8]. Unbiased proteomic analysis in cerebrospinal fluid (CSF) allows studying multiple molecular processes at the same time in patients, and it can be hypothesized that distinct patterns of protein concentrations exist in the CSF that are associated with aggregated amyloid. The first CSF proteomic studies have identified novel markers associated with AD-type dementia when comparing patients with cognitively normal controls [9–11]. Yet, not all subjects with a clinical diagnosis of AD-type dementia have aggregated amyloid, and on average, 30% of cognitively normal subjects are already in the preclinical stage of AD [3, 12, 13]. Consequently, it remains uncertain which of the previously reported markers are specific for AD pathology, i.e., aggregated amyloid. Furthermore, protein levels in CSF may depend on APOE ε4 genotype, which has been reported for beta secretase-1 [BACE1] [14] and chitinase-3-like protein 1 [YKL40] [15], both proteins associated with AD-type dementia, and so, it is plausible that APOE ε4 genotype may influence other protein markers in the CSF as well.

In this study, we used a CSF proteomic approach to test the hypothesis that protein signatures can be detected that show APOE ε4 genotype-dependent associations with AD across the cognitive spectrum.

## Methods

### Participants

We downloaded ADNI data in August 2017 from the ADNI database (all data is available at adni.loni.usc.edu), including participants from over 50 sites across the USA and Canada (www.adni-info.org). As such, the investigators within the ADNI contributed to the design and implementation of ADNI and/or provided data but did not participate in the analysis or writing of this report. Further details about ADNI are given in the “Acknowledgments” section. Study protocols were approved by the institutional review boards of all participating ADNI centers (a complete list of ADNI sites is available at http://adni.loni.usc.edu/about/centers-cores/study-sites/), and written informed consent was obtained from all participants or authorized representatives. All analyses were performed on de-identified ADNI data, and methods were carried out in accordance with the approved guidelines.

For the present study, we included individuals who had baseline CSF data available for amyloid β 1–42 and proteomics (see the next section). Aggregated amyloid in CSF was defined as having CSF amyloid β 1–42 levels below 192 pg/ml [16]. APOE genotype was assessed with two SNPs (rs429358, rs7412) that define the epsilon 2, 3, and 4 alleles, using DNA extracted by Cogenics from a 3-ml aliquot of EDTA blood. Subjects were classified according to amyloid status (normal/abnormal), APOE ε4 genotype (carrier/non-carrier), and cognitive stage as measured with the MMSE [17] (normal cognition: MMSE > 27; mild impairment: MMSE scores between 27 and 24; moderate impairment: MMSE < 24).

### CSF protein analysis

CSF samples were collected with a lumbar puncture, and samples were stored at the ADNI Core Laboratory at the University of Pennsylvania Medical Center on dry ice until further analysis. In total, 313 proteins and protein fragments were measured: 12 with ELISA, 159 with proteomics RBM, and 142 with proteomics multiple reaction monitoring (MRM) targeted mass spectroscopy (see supplementary table 1 for an overview of all included proteins). Information on protein assessment and quality control is described at http://adni.loni.usc.edu/data-samples/biospecimen-data/. For MRM, we used the finalized “Normalized Intensity” data [9], which was the result of a two-step normalization procedure of the raw peak area data to remove variability between samples processed on different days introduced by the depletion method: First, process-related bias was removed by correcting for trends when observed, by computing the predicted average log-intensity values from smoothing spline function to the CSF sample averages. For each sample at a given transition, the predicted value was subtracted from the sample average log-intensity. Second, using two regression models to model the daily sample average and the global sample average, the log-intensity values of the CSF samples after step 1 normalized were further normalized to account for the depletion day of the samples. (please see for a detailed explanation of the normalization procedure the “Biomarkers Consortium CSF ProteomicsMRM data set” in the “Data Primer” document at adni.loni.ucla.edu). All CSF protein levels were *Z*-transformed to the control group (normal amyloid, APOE ε4 non-carrier, MMSE > 27), such that negative values indicate lower and positive values indicate higher levels compared to the normal state. If peptides from the same protein showed a moderate to strong correlation (*r* > .6), we combined peptides into a composite measure by averaging their *Z* scores. This resulted in 243 protein measures tested. A subset of 114 proteins tested in the present study was also measured with tandem mass tag spectrometry in the multi-center EMIF-AD Multimodal Biomarker Discovery (MBD) study [18] which we used for replication analyses.

### Statistical analysis

*t* test, *χ*^2^, and Kruskal-Wallis tests were used to compare the subject characteristics between the AD and control groups. We compared the protein levels between subjects with AD (defined as having aggregated amyloid) and the control group, stratified for APOE ε4 genotype and cognitive stage with ANCOVAs that included age and gender as potential confounders. All statistical analyses were performed using R, version 3.2.3.

### Pathway enrichment analyses

We used the online database STRING [19] to identify enriched biological processes (based on KEGG pathways and GO biological processes) for each protein that showed significant differences with the control group. In addition, we used this database to test for pathway enrichment entering all proteins associated with a particular group at the same time.

## Results

### Sample characteristics by APOE ε4 genotype

In total, 253 individuals from the Alzheimer’s Disease Neuroimaging Initiative-1 (ADNI-1) had available baseline proteomic CSF data. Compared to the control group, APOE ε4 carriers and non-carriers with aggregated amyloid β had similar average age, level of education, and gender distributions (Table 1).

**Table 1:** Sample description

Characteristic	APOE ε4 non-carriers				APOE ε4 carriers			
	Abeta normal (control), MMSE> 27	Abeta abnormal, MMSE > 27	Abeta abnormal, MMSE 27–24	Abeta abnormal, MMSE < 24	Abeta Normal, MMSE > 27	Abeta Abnormal, MMSE > 27	Abeta Abnormal, MMSE 27–24	Abeta Abnormal, MMSE < 24
*N* (% total sample)	60 (23%)	24 (9%)	34 (13%)	9 (3%)	8 (3%)	40 (15%)	63 (24%)	23 (27%)
E2/E3	15 (25%)	3 (12.5%)	6 (18%)	0	0	0	0	0
E3/E3	45 (75%)	21 (87.5%)	28 (82%)	9 (100%)	0	0	0	0
E2/E4	0	0	0	0	0	1 (2.5%)	2 (3%)	1 (4%)
E3/E4	0	0	0	0	8 (100%)	34 (85%)	41 (68%)	13 (57%)
E4/E4	0	0	0	0	0	5 (12.5%)	20 (33%)	9 (39%)
Age	75.30 (6.44)	76.70 (4.90)	75.37 (8.10)	78.82 (5.55)	72.1 (6.43)	74.97 (6.16)	74.53 (6.88)	73.32 (8.70)
Female, *N* (%)	29 (48%)	10 (42%)	13 (38%)	4 (44%)	4 (50%)	12 (30%)	30 (48%)	8 (35%)
Education years	16.00 (2.76)	16.08 (3.32)	15.62 (3.29)	16.22 (3.23)	15.50 (2.00)	16.02 (3.22)	15.25 (2.83)	15.30 (2.67)
MMSE	29.15 (0.73)	29.21 (0.78)	25.59 (1.05)^c^	21.89 (1.05)***	29.12 (0.64)	28.73 (0.78)**	25.49 (1.11)***	21.91 (1.12)***
Amyloid β 1–42 pg/ml	245.53 (26.38)	150.5 (28.76)***	142.29 (25.06)***	148.78 (19.32)***	235.75 (22.13)	132.05 (23.19)***	131.94 (28.07)***	128.61 (23.52)***
T-tau pg/ml	63.41 (22.05)	88.12 (55.61)*	110.68 (51.83)***	149.44 (75.91)***	77.12 (19.77)	106.91 (48.96)***	120.98 (54.37)***	124.91 (51.75)***
P-tau pg/ml	20.56 (7.86)	31.33 (15.95)*	40.21 (18.04)***	49.33 (20.43)***	22 (4.41)	37.18 (14.81)***	39.84 (13.70)***	44.39 (23.78)***
APOE ε4 isoform	6.18 (2.18)	5.98 (3.26)	5.84 (2.68)	5.61 (4.44)	10.07 (5.80)^c^	11.92 (1.24)***	11.89 (1.43)***	11.63 (1.17)***

Values are mean (SD). All comparisons are made against the control group (APOE ε4 non-carriers with normal amyloid and MMSE > 27). APOE ε4 isoform is the protein fragment that is specifically encoded by the ε4 allele, average log-intensity value are provided (all non-carriers have values below the level of detection, Spellman et al., 2015)

*p < .05, **p < .01, ***p < .001

**Fig. 1:**
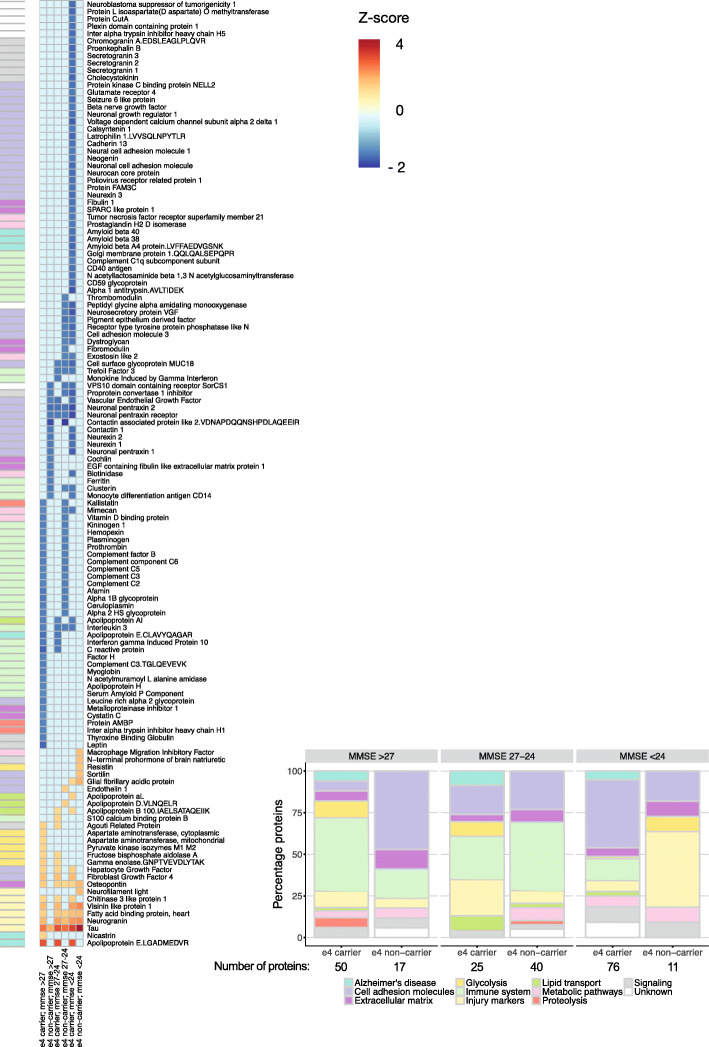
Left, heatmap of proteins associated with amyloid pathology. Columns indicate APOE ε4 carriers and non-carriers with AD according to the severity of their cognitive impairment (MMSE > 27, 27–24, or < 24). Color scale indicates the *Z* value of proteins showing a significant difference (*p* < .05) compared with the control group (APOE ε4 non-carriers with normal amyloid and MMSE > 27). Proteins are expressed as *Z*-scores using the control group as a reference and plotted when showing a significant difference (*p* < .05). Light blue indicates non-significance (*p* > .05). Right, the percentage of proteins associated with one of the 11 biological process categories. Percentages were calculated with disease stage-specific total numbers of proteins associated with abnormal amyloid, and the number of proteins is given below each disease stage. Please see supplementary table 3 for a detailed description of biological processes enriched

Figure 1 shows that proteins associated with aggregated amyloid formed distinct clusters depending on the APOE genotype and cognitive stage. In total, 129 (53%) proteins and protein fragments were associated with aggregated amyloid, with 27 (21%) proteins showing higher levels and the majority of proteins (102, 79%) showing lower levels in AD compared to controls. The large majority (90%) of proteins associated with aggregated amyloid showed expression differences with controls that depended on the cognitive stage. Tau, another major pathological hallmark for AD, was the only protein that showed higher levels in all AD subjects across the cognitive spectrum, with higher concentrations for more severe impairment, regardless of APOE ε4 status. We further observed two patterns of protein expression levels: (1) 83 of the 129 proteins (64%) had altered levels either in ε4 carriers or non-carriers; (2) 46 of the 129 proteins (36%) had altered levels in both APOE ε4 carriers and non-carriers, but in different cognitive stages.

### APOE ε4 genotype associations of proteins with aggregated amyloid β

Compared to controls, APOE ε4 carriers with normal cognition showed higher levels of nicastrin [NCSTN], which is part of the gamma secretase complex, and of a group of proteins that were associated with glycolysis (Fig. 1, first column; supplementary table 1). Carriers further showed higher levels of markers known to increase with neuronal injury (neurogranin [NRGN]; fatty acid-binding protein, heart [FABP3]; visinin-like protein-1[VILIP1]; YKL40), growth factors fibroblast growth factor-4 [FGF4], and hepatocyte growth factor [HGF] [20]. These proteins were also higher in subjects with mild and moderate cognitive impairment. Furthermore, a large group of proteins had lower levels in AD, including immune system-related complement factors (C2, C3, C5, C6, factor-B [CFB], factor-H [CFH]) and factors that interact with the complement system (plasminogen [PLG], prothrombin [F2], serum amyloid P component [APCS], and C-reactive protein [CRP]). Subjects with moderate cognitive impairment showed lower levels of proteins that were mostly associated with cell adhesion-related processes (Fig. 1, fifth column), including markers functionally associated with “transsynaptic signaling”(e.g., cadherin-13 [CDH13], neogenin [NEO1], neural cell adhesion molecule-1 [NCAM1], neuronal cell adhesion molecule [NRCAM]), “peptide neurotrophin signaling” (chromogranin-A [CHGA], proenkephalin-B [PDYN], secretogranin-2 [SCG2], proSAAS [PCSK1]), and “GPCR signaling”(glutamate receptor-4 [GRM4], latrophilin-1 [ADGRL1]) [21]. The top pathways enriched in KEGG for proteins associated with aggregated amyloid were “complement and coagulation cascades” for subjects with normal cognition; no enrichment was observed in mild impairment, and for moderate cognitive impairment “cell adhesion molecules” (Table 2).

**Table 2:** Summary of pathways enriched in KEGG of proteins associated with aggregated amyloid according to APOE ε4 genotype

Cognitive stage	APOE ε4 carriers			APOE ε4 non-carriers		
	Pathway enriched	pFDR	Proteins	Pathway enriched	pFDR	Proteins
MMSE > 27	Complement and coagulation cascades	8.20E−12	**C2**, **C3**, C5, C6, CFB, **CFH**, F2, **KNG1**, PLG	Cell adhesion molecules	0.000165	CADM3’, **NRXN2**, **NRXN1**, **CNTN1**, **CNTNAP2**
MMSE 27–24	No enrichment detected	n.a.	n.a.	Complement and coagulation cascades	8.61E−13	C2, C3, **C5**, C6, CFB, F2, **KNG1**, PLG
MMSE < 24	Cell adhesion molecules	8.44E−09	CADM3’, **CNTN1**, **CNTNAP2**, **NCAM1**, **NEGR1**, **NEO1**, **NRCAM**, **NRXN1**, **NRXN2**, PVRL1’	No enrichment detected	n.a.	n.a.

Please note that APOE and APP were excluded from enrichment analyses. Bold font indicates concordant difference compared to controls observed in EMIF-AD MBD; apostrophe (’) indicates not available in independent cohort

*n.a* not applicable, *MMSE* Mini-Mental State Examination, *FDR* false discovery rate

### APOE ε4 non-carrier associations of proteins with aggregated amyloid β

APOE ε4 non-carriers with aggregated amyloid showed a different proteomic profile than APOE ε4 carriers, in the type of proteins expressed and/or the cognitive stage of expression. Non-carriers with normal cognition showed lower levels of a large group of proteins associated with cell adhesion processes compared to the control group (Fig. 1; supplementary table 2). A subset of these proteins included synaptic markers contactin-1 [CNTN1], neurexin-1 [NRXN1] and neurexin-2 [NRXN2] that were associated with “transsynaptic signaling” [21], and the neuronal pentraxin receptor [NPTXR]. In this stage, only tau showed higher levels. APOE ε4 non-carriers with mild impairment showed lower levels of complement-related proteins, which overlapped with the complement proteins that showed lower levels in APOE ε4 carriers with normal cognition. Further, alterations observed in non-carriers with mild impairment were higher levels of a wide range of neuronal injury markers (NRGN, FAPB3, VILIP1). APOE ε4 non-carriers with moderate cognitive impairment also had higher levels of glial fibrillary acidic protein [GFAP], neurofilament light [NFL], resistin [RETN], and macrophage migration inhibitory factor [MIF]. These proteins did not show a clear association with a shared biological pathway (Fig. 1, sixth column), but might be related to inflammatory responses. In addition, sortilin [SORT1] levels were higher in these subjects. SORT1 has several functions and is involved in APP processing [22]. No proteins showed lower levels in this stage, but it should be noted that this group had a small sample size, which may have limited statistical power. The top pathways enriched in KEGG for proteins associated with aggregated amyloid in APOE ε4 non-carriers were “cell adhesion molecules” for subjects with normal cognition and “complement and coagulation cascades” for subjects with mild impairment (Table 2).

### APOE ε4 effect on amyloid processing in asymptomatic subjects with normal amyloid β

**Fig. 2:**
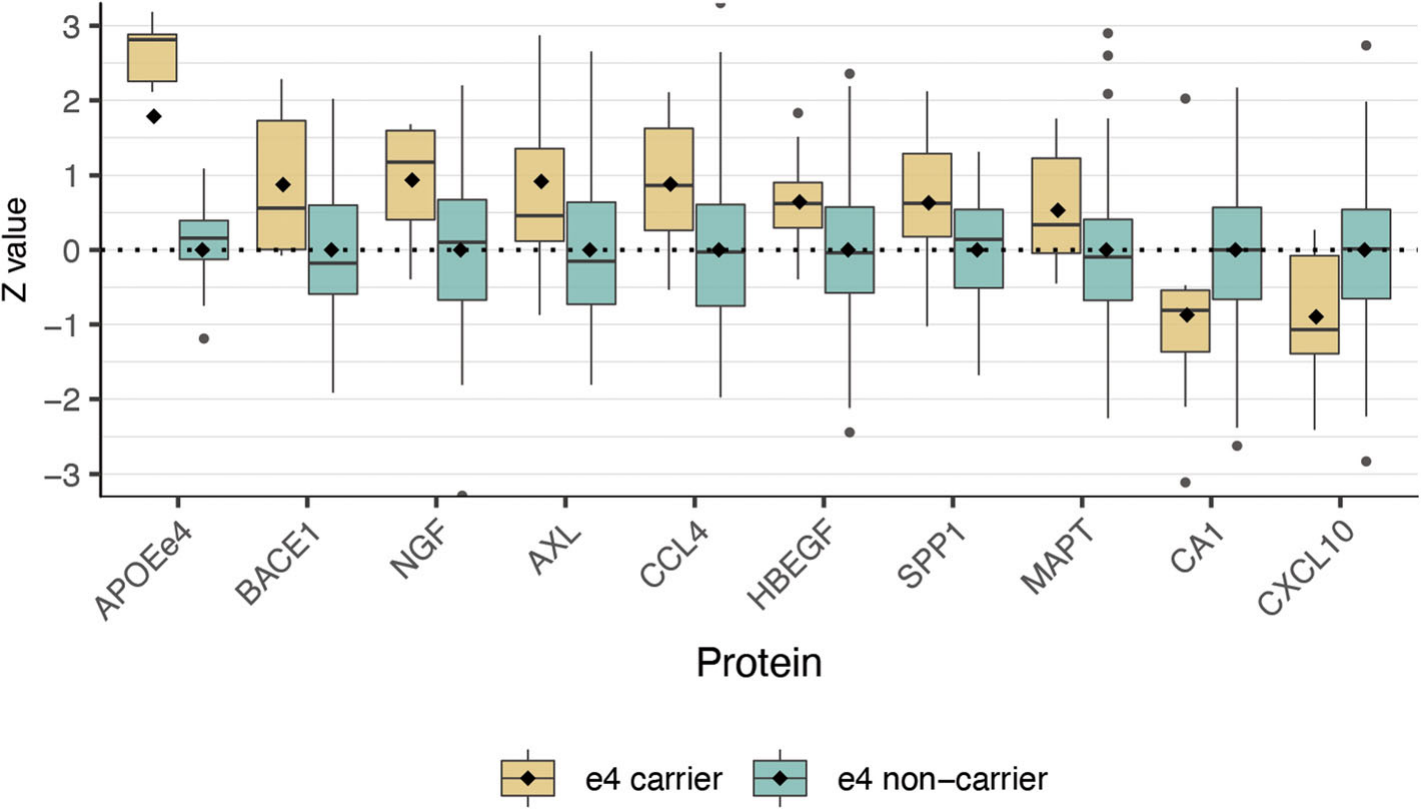
Proteins associated with APOE ε4 carrier status in subjects with normal amyloid. *Z*-scores are plotted for proteins that were different between subjects with APOE ε4 (in brown) and normal amyloid and normal cognition (MMSE > 27) compared to the control group (i.e., in blue). All values are standardized according to the control group (i.e., APOE ε4 non-carriers with normal amyloid and MMSE > 27)

We further explored whether protein differences could be observed in APOE ε4 carriers with normal amyloid β and cognition, as these subjects are at increased genetic risk of developing amyloid pathology [4], and so, for these subjects, proteomic alterations may indicate very early pathological changes associated with AD. Injury markers were normal in these subjects. Compared to the control group, ten proteins (APOE ε4 fragment, tau [MAPT], BACE1, β-nerve growth factor [NGF], macrophage inflammatory protein-1β [CCL4], osteopontin [SPP1], AXL receptor tyrosine kinase [AXL], heparin-binding EGF-like growth factor [HBGF], carbonic anhydrase-1 [CA1], interferon gamma-induced protein-10 [CXCL10]) showed altered levels (Fig. 2). The top KEGG pathway enriched was the Toll-like receptor signaling pathway (pFDR = 0.00033; including SPP1, CXCL10, and CCL4).

### Replication in the independent EMIF-AD MBD cohort

**Fig. 3:**
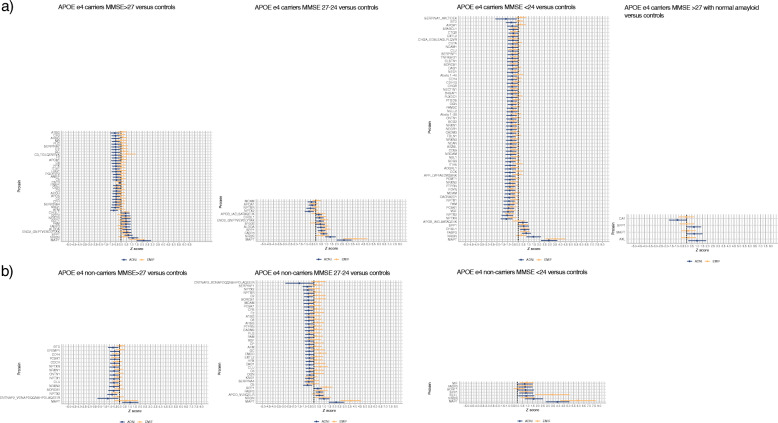
Effect size plots for 114 proteins measured in both ADNI and EMIF-AD MBD studies. **a** Effects for protein differences of individuals with abnormal amyloid carrying and ≥ 1 APOE ε4 allele against controls (i.e., APOE ε4 non-carriers with normal amyloid and MMSE > 27) according to the severity of their cognitive impairment (MMSE > 27, 27–24, or < 24), and at the far left for APOE ε4 carriers with normal amyloid and MMSE > 27. In blue, effect sizes and 95%CI for ADNI; in orange, for EMIF-AD MBD. **b** Effects for protein differences of individuals with abnormal amyloid carrying without an APOE ε4 allele against controls (i.e., APOE ε4 non-carriers with normal amyloid and MMSE > 27) according to the severity of their cognitive impairment (MMSE > 27, 27–24, or < 24). In blue, effect sizes and 95%CI for ADNI; in orange, for EMIF-AD MBD

Finally, we performed replication analyses in the independent EMIF-AD MBD cohort by comparing the concordance of observed group differences compared to controls. Overall, individuals in EMIF-AD MBD were younger than in ADNI and showed lower levels of education (supplementary table 4). APOE ε4 carriers with mild impairment were more often female in EMIF-AD MBD (54%) than in ADNI (30%). Of the 136 proteins/fragments that showed an association with amyloid aggregation in the carrier or non-carrier groups, 114 were also measured in the EMIF-AD MBD, and across all contrasts, 80% showed concordant group differences compared to controls. Within in the APOE ε4 carriers, individuals with mild and moderate cognitive impairment showed high concordances in group differences of respectively 93% of 13 overlapping proteins and 95% of 63 overlapping proteins (Fig. 3; supplementary table 1), and individuals with normal cognition showed moderate concordance of 58% of 40 overlapping proteins. APOE ε4 carriers with normal cognition and normal amyloid showed concordance of 100% of 4 overlapping proteins. In the APOE ε4 non-carriers, individuals with mild impairment showed a low level of concordance of 28% of 36 overlapping proteins, and individuals with normal cognition and moderate cognitive impairment showed high concordance (respectively 87% of 15 overlapping proteins and 100% of 7 overlapping proteins; Fig. 3; supplementary table 2).

## Discussion

### Summary

In this study, both APOE ε4 carriers and non-carriers with abnormal CSF amyloid β 1–42 levels showed alterations of large groups of proteins involved in neuronal injury, complement and inflammatory processes, and cell adhesion processes, but in a different temporal ordering. APOE ε4 carriers showed altered protein levels of complement related proteins in the normal cognition stage, while lower levels of proteins associated with cell adhesion and synaptic signaling were found in cognitive impairment stages. Non-carriers with aggregated amyloid showed a reversed temporal ordering of these processes with proteins involved in cell adhesion processes showing altered levels in cognitively normal subjects, which was followed by alterations in complement-related proteins in cognitive impairment stages. These results suggest that subjects with AD may require specific treatment tailored to their APOE genotype and degree of cognitive impairment.

### CSF proteome signatures associated with APOE ε4

APOE ε4 carriers with normal cognition showed lower levels of complement-related proteins C2, C3, C5, C6, CFB, CFH, PLG, F2, APCS, and CRP. The complement system is a major part of the innate immune system, and its classical activation pathway can be directly triggered by amyloid aggregates [23, 24]. Previous studies investigating complement-related protein concentrations in CSF have, however, reported divergent results with higher concentrations in AD-type dementia patients [25–27] and also lower concentrations in AD-type dementia patients [28] and in subjects with mild cognitive impairment who showed cognitive decline at follow-up [27]. Our results suggest that levels may be altered in different cognitive stages according to APOE genotype, with ε4 carriers showing more extensive complement involvement in the cognitively normal stage, whereas non-carriers showed alterations in the mild impairment stage. Possibly, this observation reflects that the apoE4 protein enhances complement activation in the presence of aggregated amyloid β [29]. However, only a subset of these proteins (KNG1, CFH, C2) showed similar differences in the replication cohort, and these were less pronounced. The replication cohort further showed contrary group differences compared to ADNI for C6, CFB, F2, and PLG in both APOE carriers with normal cognition and in APOE non-carriers with mild impairment. This suggests that the different proteomic techniques may differ in sensitivity to measure these proteins accurately, and future research should further investigate this issue by comparing both techniques within the same cohort. At this point, we can only speculate as to how reduced levels of complement proteins in CSF can be interpreted. Lower protein concentrations may reflect binding of complement proteins to pathogen surfaces, possibly to tag these for phagocytosis [23], and the presence of complement proteins in amyloid plaques seems to support this explanation [23, 24, 30]. Alternatively, lower levels of complement proteins could point towards decreased production, which seems to be in line with the observation that regulators of complement activation like CFH also showed lower levels in these subjects. Furthermore, carriers in the moderate cognitive impairment stage showed lower levels than controls of complement C1q subcomponent subunit-B [C1QB] and CD59, which was also observed in the replication cohort. C1q can directly bind to amyloid β fibrils, which can lead to activation of C1 as well as C3 [24, 31]. Whereas C3 is associated with several pathways of the complement system, C1QB is specific for classical complement activation [23, 24]. The involvement of different complement proteins according to cognitive stage suggests that triggers of the complement system might exist that depend on the level of neuronal injury and/or the degree of amyloid fibril formation. Future research should further study how complement levels change longitudinally during the development of AD, and how these processes depend on APOE genotype.

Alterations of complement protein concentrations were accompanied by a range of inflammatory markers in APOE ε4 carriers, some of these showing altered levels in carriers who had still normal amyloid β levels, suggesting that inflammation processes may play a role in the development in AD before amyloid aggregation becomes manifest in CSF. Some of these markers have been associated with microglia dysfunction or response associated with neurodegeneration (AXL, SPP1, FABP3) [32, 33] and reactive astrocytes (CCL4, S100 calcium binding protein B [S100B], YKL40, GFAP) [34]. In APOE ε4 non-carriers, most of these protein levels were similar to controls, except for inflammation markers SPP1, FABP3 (also in the replication cohort), and GFAP that were higher in more severe stages of cognitive impairment. Together, these results support the notion that inflammation plays an important role in AD [35], and we further show that the timing of involved inflammation processes seem to depend on APOE genotype. It is conceivable that these differences reflect apoE isoform-specific interactions with astrocyte and microglia functioning [32, 33, 36, 37].

APOE ε4 carriers with normal amyloid β showed higher levels of BACE1, which is the secretase that initiates amyloidogenic processing of APP [38]. This suggests that increased APP processing might be a pre-amyloid event [39]. Tau levels were also already increased, although lower than in carriers with normal cognition who showed aggregated amyloid. Cognitively normal APOE ε4 carriers with aggregated amyloid further showed higher levels of proteins associated with glycolysis, which was also observed in the replication cohort. High levels of proteins involved in glycolysis have previously been reported in brain pathology studies in early stage AD [40]. APOE ε4 has been associated to dysfunction of mitochondria [41], and so increased levels of glycolysis may indicate compensation for mitochondrial dysfunction [41–44]. In the mild impairment stage, APOE ε4 carriers showed lower levels of a small group of cell adhesion molecules, and substantially more cell adhesion proteins showed lower levels in the moderate impairment stage, among which proteins associated with synapse development (NPTXR, NRCAM, NEO1, NCAM1, CDH13), which was also observed in the replication cohort [21]. In addition, proteins that showed lower concentrations than controls were associated with presynaptic dense core vesicles (CHGA, secretogranin-3 [SGC3], voltage-dependent calcium channel subunit alpha-2 delta-1 [CACNA2D1], PDYN, CDH13, SPARC-like protein 1[SPARCL1], alpha-1 antitrypsin [SERPINA1]) [45]. These proteins are associated with peptide neurotrophin signaling. Since synapse loss correlates well with cognitive decline [46], and in more severe cognitive impairment stages these proteins showed lower levels, also in the replication cohort, this might indicate impaired synaptic functioning. However, APOE ε4 non-carriers showed normal levels of the majority of these proteins despite the same stage of cognitive impairment and similar levels of neuronal injury markers.

### CSF proteome signatures associated with aggregated amyloid β in APOE ε4 non-carriers

Non-carriers with aggregated amyloid and normal cognition showed lower levels of presynaptic proteins (NRX1 and NRX2), proteins involved intracellular trafficking (vacuolar protein sorting 10 [VPS10], domain-containing receptor SorCS1 [SORCS1]), and neuronal pentraxins [47], suggesting alterations in the presynaptic cell structure may be an early event in AD for subjects lacking the ε4 allele. These differences with controls were also observed in the replication cohort, albeit less pronounced. In particular, SORCS1 stands out in this group, as this gene has been associated with an increased risk for AD [48], and this protein plays a key role in intracellular sorting and trafficking of proteins, including APP, neuronal pentraxins, and NRX1 and NRX2 [48–50]. This leads us to propose that the lower levels we observed presently in ε4 non-carriers with still normal cognition might reflect disruption of these cellular transport mechanisms and subsequent failure of intracellular processes such as protein recycling, exocytosis, or autophagocytosis. Levels of the synaptic proteins were low in the mild impairment stage, and in that stage, additional proteins associated with cell adhesion processes, like, e.g., cell adhesion molecule-3 [CADM3] also showed lower levels. The replication cohort showed, however, higher levels for those proteins. It is unclear whether this reflects differences in disease stage, or age, as the replication cohort was younger. Differences in the results between the cohorts may also reflect the differences between proteomic techniques. More research is needed that use the same proteomics technique in larger samples of non-carriers with mild impairment to further investigate this issue. Another finding was that we observed higher NFL levels in non-carriers in both cohorts. Higher levels of CSF NFL indicate axonal injury, and such higher levels have been associated with neurodegenerative processes in several neurological disorders [51].

### Strengths and limitations

A potential limitation of this study is that the interpretation of higher and lower levels of proteins measured in CSF in terms of activated biological processes is not always straightforward. However, interpretations for some proteins, such as amyloid β and tau, have been well established through associations with histopathological measurements in post-mortem research [52] and/or with PET imaging [53]. Still, 80% of the associations for proteins that were also measured in an independent replication cohort showed group differences in a similar direction, but this depended on the specific group studied, and small sample sizes of these subgroups (particularly so for the non-carriers with MMSE < 24 and the carriers with normal amyloid and MMSE > 27) may have limited the statistical power to observe significant results. Therefore, more studies are needed to replicate these findings in larger samples. Nevertheless, our results may be useful to select proteins for further detailed studies, as these seem to be involved in AD pathology in vivo. Another point of note is that proteins measured in ADNI were specifically selected based on their association with biological processes relevant for AD. As such, pathway enrichment analyses should not be seen as discovery of new processes involved, but rather were meant to aid interpretation of the results. The enriched processes associated with abnormal amyloid where specific for clinical stage and genotype, and it is unlikely that enrichment of these processes simply reflect the processes associated with the proteins that were selected in this study, because the background set of proteins was the same each analysis. Untargeted proteomic techniques should be used in order to discover involvement of potential other pathways that may be associated with AD in an APOE dependent way. Another point of note is that the control group contained more E2 carriers than the APOE ε4 carriers, and consequently, differences between these groups may also contain E2-specific effects, which may not directly reflect amyloid abnormality. Furthermore, APOE ε4 carriers with MMSE > 27 were less often ε4 homozygous than carriers with MMSE < 27 and abnormal amyloid, and such dose effects may have contributed to the different sets of proteins observed to be related with abnormal amyloid between these stages. Future studies should aim to collect larger samples of individuals in order to determine dosage effects on the CSF proteome.

Furthermore, we have operationalized disease severity in our sample based on the MMSE, which is a screening tool. An alternative approach would have been categorization based on syndrome diagnosis, but a drawback of that approach is that individuals with normal cognition, MCI, and dementia can have the same MMSE. At this point, no tools exist that accurately delineates disease severity in a non-demented population, and future research should focus on developing tools that are sensitive to cognitive impairment in pre-dementia stages of AD. Another potential limitation is that we labeled proteins based on the top pathways found, and although this simplifies the interpretation of the results, this approach does not take into account the notion that proteins could be involved in multiple biological processes. In addition, in the present study, we defined AD based on abnormal CSF amyloid levels, because (as of yet) the majority (97%) of subjects did not have pathological data available, which can be seen as a limitation of this study. Still, it has been shown previously that amyloid biomarker values correlate with histopathological measures for amyloid plaques [52]. Using biomarkers to define AD can also be regarded to be a strong aspect of our study. This way, we avoided potential biases in our results that may arise when defining groups based only on clinical characteristics, as clinical features do not always accurately reflect the underlying pathology. Finally, although our results suggest that several processes associated with aggregated amyloid might be transient, further longitudinal CSF proteomic studies are required to investigate these dynamics in more detail.

## Conclusions

In conclusion, we found CSF proteomic signatures that were associated with aggregated amyloid β and were dependent on APOE ε4 genotype and cognitive stage. An implication of our results is that AD subjects may require treatments tailored to APOE genotype and that clinical trials may need to consider APOE ε4 dependent endpoints in CSF.

## Supplementary information

**Additional file 1.** Supplementary tables.

## Data Availability

Data collection and sharing for this project was funded by the Alzheimer’s Disease Neuroimaging Initiative (ADNI) (National Institutes of Health Grant U01 AG024904) and DOD ADNI (Department of Defense award number W81XWH-12-2-0012).

## Abbreviations

CSF: Cerebrospinal fluid
APOE: Apolipoprotein E
AD: Alzheimer’s disease
MMSE: Mini Mental-State Examination
BACE1: Beta secretase 1
YKL40: Chitinase-3-like protein 1
ADNI: Alzheimer’s Disease Neuroimaging Initiative
RBM: Rules-based medicine
MRM: Multiple reaction monitoring
NCSTN: Nicastrin
NRGN: Neurogranin
FABP3: Fatty acid-binding protein, heart
VILIP1: Visinin-like protein-1
FGF4: Fibroblast growth factor
HGF: Hepatocyte growth factor
C2, C3, C5, C6: Complement factors 2, 3, 5, 6
CFB: Complement factor B
CFH: Complement factor H
PLG: Plasminogen
F2: Prothrombin
APCS: Serum amyloid P component
CRP: C-reactive protein
CDH13: Cadherin-13
NEO1: Neogenin
NCAM1: Neural cell adhesion molecule-1
CHGA: Chromogranin-A
PDYN: Proenkephalin
SCG2: Secretogranin-2
PCSJ1: proSAAS
GRM4: Glutamate receptor-4
ADGRL1: Latrophilin-1
CNTN1: Contactin-1
NRXN1: Neurexin-1
NRXN2: Neurexin-2
NPTXR: Neuronal pentraxin receptor
GFAP: Glial fibrillary acidic protein
NFL: Neurofilament light
RETN: Resistin
MIF: Macrophage migration inhibitory factor
SORT1: Sortilin
NGF: β-Nerve growth factor
CCL4: Macrophage inflammatory protein-1β
SPP1: Osteopontin
AXL: AXL Receptor tyrosine kinase
CLSTN1: Calsyntenin-1
CXCL9: Monokine induced by gamma interferon
CA1: Carbonic anhydrase-1
CXCL10: Interferon gamma-induced protein-10
S100B: S100 calcium-binding protein B
SPARCL1: SPARC-like protein 1
SERPINA1: Alpha-1 antitrypsin
VSP10: Vacuolar protein sorting 10
SORCS1: Domain-containing receptor SorcCS1
CADM3: Cell adhesion molecule-3
